# A Multi-Stage Planning Method for Distribution Networks Based on ARIMA with Error Gradient Sampling for Source–Load Prediction

**DOI:** 10.3390/s22218403

**Published:** 2022-11-01

**Authors:** Sheng Yan, Minqiang Hu

**Affiliations:** School of Electrical Engineering, Southeast University, Nanjing 210096, China

**Keywords:** distribution network, source–load prediction, multi-stage planning method, ARIMA, error gradient sampling, data acquisition, precise control, sensory feedback

## Abstract

As the scale of distributed renewable energy represented by wind power and photovoltaic continues to expand and load demand gradually changes, the future evolution of the smart distribution network will be directly driven by both distributed generation and user demand. The smart distribution network contains a wide range of flexible resources, and its flexibility and uncertainty will bring great challenges to grid data acquisition and control feedback. To adapt to the precise control and feedback of smart distribution network access equipment under the high proportion of new energy access and to ensure the safe operation of the system, it is urgent to accelerate the study of the evolution of the future distribution grid based on the existing distribution grid. Hence, a multi-stage planning method for distribution networks based on source–load prediction is proposed in this paper. Firstly, a distribution network source–load prediction method based on the autoregressive integrated moving average model (ARIMA) and error gradient sampling is proposed, using ARIMA to predict the scale of source–load development and error gradient sampling based on the generation of source–load scenarios with error intervals. K-means is further used for scenario reduction, to explore multiple operating scenarios of China’s distribution network source–load, and the unit’s output forecast interval and load demand from 2021 to 2030 for typical regions are derived using rolling forecasts by combining the unit’s output, end-demand and clean energy share over the years. Secondly, the planning model of distribution grid evolution in different stages is constructed to analyze the future evolution form of the distribution grid considering the distribution network’s load cross-section, respectively, and to provide a development path reference for the future construction of distribution grid form in China.

## 1. Introduction

With the broad access to new energy sources such as wind power and photovoltaic, and the development of customer-side electricity load, the increase in its controllability has put higher requirements on the operation mode and structure form of the distribution network. The distribution network is no longer a passive network that simply receives and distributes electricity but a primary support platform that carries a large amount of distributed energy access and supports electricity interaction between users in production and sales. Due to the significant uncertainty and volatility of distributed clean energy sources such as wind power and photovoltaic, it is essential to consider not only the planning constraints of component and equipment investments but also the operation of the distribution network under different distributed renewable energy output scenarios in the distribution network planning [1,2]. Not only the traditional investment in transformers and distribution lines should be considered, but also the allocation and utilization of flexible regulation resources such as energy storage and user-side controllable loads to enhance the flexibility of the distribution network’s operation, realize the consumption of large-scale distributed renewable power, and help the whole power system achieve its energy transformation goals [3].

The distribution network is the most active component of the grid in the current technological innovation, and its participation in grid operation is a key part of the evolution of the traditional grid to the smart grid. The widespread access to distributed energy sources such as distributed generation, energy storage, and customer-side controllable resources makes the distribution system no longer a sourceless and passive receiving network in the traditional sense. To achieve effective management and overall integration of distributed energy, energy management concepts and institutions with different coverage and distinct hierarchical characteristics such as microgrids, community microgrids [4], and active distribution networks [5] have emerged. With the coupling characteristics and complementary potential of different energy forms, the active interaction between users and the grid is also gradually emerging. Most studies of transmission and distribution networks mostly consider network planning problems under probabilistic scenarios, focusing on the construction of network planning models, and the uncertainty of source and load is mostly quantified in a probabilistic manner and transformed into deterministic scenarios. Planning studies for distribution networks have focused on the consideration of the flexibility of distributed resources in the network, starting from a single aspect such as the flexibility of source–load resources [6], demand response uncertainty [7], the controllability/uncontrollability of the grid [8,9], and the proactivity/passivity of system control [10,11,12], to plan the expansion of the grid or equipment of the distribution network. However, the intersection of many factors will undoubtedly further complicate the analysis and planning of future transmission and distribution systems. Therefore, in order to cope with the randomness and diversity of regional growth of source–loads in distribution networks, some studies have started to partition the distribution networks and plan the networks considering multiple factors on the basis of partitioning. A GIS-based dynamic planning method for distribution networks was proposed in [13], and the problem scale was simplified by the established spatio-temporal load distribution model under clustered partitioning. The cost of network planning and construction, operation and maintenance costs can be effectively reduced. The overall framework system and planning process of territorial spatial planning was analyzed in [14], a distribution grid planning process adapted to territorial spatial planning was proposed, and the spatial layout of power facilities in distribution grids was studied. The above studies mostly focus on the spatial layout of the distribution network and the cross-influence of multiple types of factors for the medium and long-term line and equipment planning of the distribution network, but with the large-scale new energy access to the distribution network and the increasing flexibility of the load, it is necessary to take into account the changing trend of the source and load and the scale of development, and realize the multi-stage planning of the distribution network based on the dual-drive digital model. Distribution network planning that has been considered data-driven or data-mode dual-driven mostly considers a single power structure or load type by mining short-term and medium-term data on source and loads as data input for the generation of future sourced load scenarios [15,16,17]. The problem of load data mining in the field of distribution network planning was addressed in [18], a model for air conditioning load mining and demand-side response evaluation was established, and a distribution network expansion planning model was further proposed to optimize the distribution network planning scheme on the basis of fully exploiting the potential of customer demand response. In [19], a data-driven distribution network planning and investment decision method based on deep learning was proposed to construct a distribution network investment return model and migration learning to solve the problem that deep learning is difficult to use effectively in the case of small samples. Although the above studies consider the development trend prediction of source–load big data but failed to analyze the evolution of the long-term scale source–load development scale trend.

In addition to models and forecasting methods, smart grid optimization techniques can also provide effective assurance of planning results in distribution network planning. The optimal configuration of systems and equipment for smart grids can also effectively help users interact with the grid for accurate data and reliable information, enhancing efficient interaction between the grid and users [20]. At this stage, optimization algorithms for distribution network planning mainly contain particle swarm, heuristic, Benders, and other optimization algorithms [21,22], Using such methods, a two-level optimization search of the planning model is performed to find the optimal planning solution. In [23], a distribution network zoning planning technique based on an improved K-means clustering algorithm for overseas less developed cities was proposed, which can effectively solve the problems of chaotic distribution network planning and uneven distribution of load points in overseas less developed cities. In summary, the exact solution of the planning model can now be achieved by selecting a suitable optimization algorithm for optimal planning of the power grid. In this paper, the MINLP algorithm in GAMS is used to optimally solve the proposed planning model, and the results show that the optimization algorithm can also achieve the exact optimization and solution of the model.

In summary, the uncertainty of source–load is the premise of all factors. The uncertainty of source–load development is quantitatively analyzed to predict its future scale trend. Based on this, the phased planning of the transmission network and distribution network is carried out, and the morphological evolution route of the power grid is finally obtained. There is not enough research in this area. Therefore, a multi-stage planning route for future distribution networks based on the prediction of future distribution network source–load morphology is investigated in this paper. Firstly, the morphological characteristics of distributed generation sources and multiple loads in different stages of the distribution network are analyzed as the basis for source–load forecasting in the distribution network. Secondly, ARIMA forecasting and error gradient sampling methods are used to make multi-year rolling forecasts of source–load data in typical areas of Jiangsu Province to form the source–load variation trends and load deviation intervals from 2020 to 2030. Then, multi-stage distribution network planning models are constructed. Finally, based on the minimum operation scenario of source–load prediction, the changing trend of source–load under different time sections is considered, and the morphological changes in the distribution network under different time nodes are analyzed and elaborated based on the source–load prediction data of Jiangsu province as input, considering the access of electric vehicles, hot and cold equipment, etc., and the morphological evolution route of the future distribution network is derived.

## 2. Morphological Evolution Mechanism Based on the Source–Load Prediction of Distribution Network

**Figure 1:**
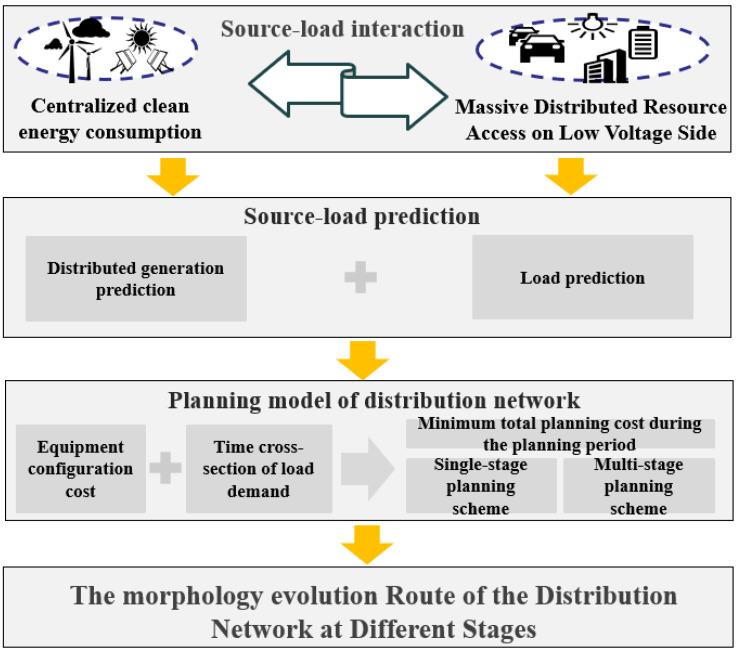
Analysis framework of multi-stage morphological evolutionary route of distribution network.

With the continuous development and construction of new power systems, there will be a large number of new energy sources connected to the distribution network in the future, and the uncertainty of new energy sources will have a greater impact on the power grid. At the same time, the scale and type of customer-side load will gradually expand from the original single fixed load to multiple flexible loads. The source–load situation of the distribution network will develop from “source follows load” to “load follows source.” Therefore, the morphological evolution of the distribution network will be mainly reflected in the increase, decrease, and expansion of grid power and customer-side equipment. In order to cope with the impact of the uncertainty of the extensive access of new energy sources on the distribution network and the interaction of multiple flexible loads on the customer side, it is necessary to analyze and predict the future source–load trend more accurately, so as to reduce the impact of source–load fluctuations on the distribution network morphology in a long-term scale. Based on the results of different types of load forecasting in typical areas, it is also necessary to consider the actual situation in different areas of the distribution network, including the development of the urban residential population, the investment and expansion of factories and shopping malls, and the growth of diversified loads and other time section nodes. It is necessary to consider the above factors, construct the planning model of the power grid, and form the optimal evolution route of the distribution network with the objective of minimizing the total planning cost during the planning period of the distribution network. Therefore, the framework of morphological evolution analysis of the distribution grid at different stages is shown in Figure 1.

## 3. Prediction of Source–Load Morphology in Distribution Networks Based on ARIMA and Error Gradient Sampling

The planning elements of the future distribution network will be richer, and various types of sources and loads will have greater variability in physical properties and dynamic characteristics. In particular, access to distributed generation sources, energy storage, electric vehicles, and other devices often has a strong degree of freedom, which leads to a significant increase in the differences and uncertainties of individual user characteristics, which not only makes it difficult to carry out uniform metrics, but also greatly increases the difficulty of analysis and prediction. In this regard, a specific analysis of various source and load planning elements is needed to condense the common features of a large number of source–load responses, while distinguishing the typical individual differences between different sources and loads.

In the analysis of the morphological evolution of the distribution network, it is necessary to take the trend of the evolution of the unsupported source–load morphology of the distribution network as the premise, while there are more types of source–loads in China’s distribution network. In the prediction and analysis of the source–load morphology, it is necessary to effectively grasp the trend of the source–load development and dig into the trend and periodicity of the source–load evolution on the basis of the current stage of the source–load development state. The ARIMA prediction model can exploit the trend of the series to be predicted on the basis of the existing time series data. Compared with the general prediction methods, its model is simple and can effectively take into account the smoothness of time series data to accurately predict the data development trend. The source–load growth rate is introduced to dynamically correct the error interval range of adjacent moments in the error gradient sampling process, and then multiple load scenarios are derived. Based on the load scenarios, the ARIMA model is used to make rolling forecasts of power sources, which eventually form multi-class source–load prediction scenarios, combined with K-means clustering to reduce the scenarios, and then derive the evolution curve of the source–load development trend.

### 3.1. The Source–Load Prediction Model Based on ARIMA

ARIMA is a time series forecasting analysis method that combines the autoregressive model and moving average model to form ARMIA(*p*, *d*, *q*) model. The input data, i.e., source–load historical data, are transformed into smooth data by difference, and then the dependent variable is regressed only on its own lagged value and the present and lagged values of the random error term to derive the final source–load forecasting results, where *p* is the number of autoregressive terms, *q* is the number of moving average terms, and *d* is the number of differences (order) made to make it a smooth series.

The smoothness of the source–load data from the input history is first tested using the difference method, which is the process of converting non-smooth data to smooth by removing its non-constant trend. Firstly, the source–load data of the input history is smoothed using the difference method defining the source–load series of the *t*th day of the history as *x_t_* and the difference process as the difference between the time series at *t* and *t* − 1 moments, as shown in Equation (1).

(1) $$\Delta x_{t}=x_{t}-x_{t-1}$$

where $\Delta x_{t}$ is the first-order difference of the time series at moment *t*. The second-order difference is performed on the basis of the first-order difference to observe whether the data are smooth or not.

(2) $$\Delta (\Delta x_{t})=\Delta x_{t+1}-\Delta x_{t}$$

where $\Delta (\Delta x_{t})$ is the second-order difference of the time series at moment *t*.

The difference method is used to determine the difference order d, when the time series tends to be smooth. On this basis, the ARIMA model is constructed, and the orders *p* and *q* are determined at the same time. The autoregressive order *p* and the moving average order *q* need to be determined in the modeling process of the ARIMA model. *p* and *q* are usually selected using the autocorrelation function (*ACF*) and the partial autocorrelation function (*PACF*). *ACF* is used to describe the linear correlation between the predicted and historical values of the time series, and *PACF* is used to describe the linear correlation between the predicted values of the time series expected past observations given intermediate observations. The formulas for *ACF* and *PACF* are as follows.

It is known that the input sequence *X* = {*x*_1_, *x*_2_, *x_t_*,..., *x_n_*}, whose mean is denoted by *u* as shown in Equation (3), and variance is denoted by σ^2^ as shown in Equation (4).

(3) $$u=\frac{1}{n}\sum_{i=1}^{n}x_{i}$$

(4) $$\sigma^{2}=\frac{1}{n}\sum_{i=1}^{n}{\left(x_{i}-u\right)}^{2}$$

For two different series *X* and *Y* of equal length, the covariance can be used to portray their correlation, and the specific covariance formula is as follows.

(5) $$cov\left(X,Y\right)=\frac{1}{n}\sum_{i=1}^{n}\left(x_{i}-u_{x})(y_{i}-u_{y}\right)$$

where *u_x_* and *u_y_* denote the mean values of series *X* and *Y*, respectively. The larger the value of the covariance *cov* (*X*, *Y*), the stronger the correlation between the sequences *X* and *Y* (positive correlation when greater than 0, negative correlation when less than 0). Similarly, for the sequence *X*, the corresponding serial self-covariance is calculated based on the number of lags *k* of the sequence, i.e.,

(6) $$c_{k}=\frac{1}{n-k}\sum_{i=k+1}^{n}\left(x_{i}-u)(x_{i-k}-u\right)$$

According to *c_k_*, the autocorrelation coefficient *ACF* can be obtained as:

(7) $$ACF(k)=\frac{c_{k}}{c_{0}}=\frac{n}{n-k}\frac{\sum_{i=k+1}^{n}\left(x_{i}-u)(x_{i-k}-u\right)}{\sum_{i=1}^{n}\left(x_{i}-u)(x_{t}-u\right)}$$

The partial correlation coefficient *PACF* of the series can be obtained from the *c_k_* and Toeplitz matrix as:

(8) $$PACF(k)={\left(R^{-1}r\right)}_{k}^{\left(k\right)}$$

where *R* is the Toeplitz matrix, which can be expressed by Equation (10), and *r* is the autocorrelation vector, which can be expressed by Equation (11).

(9) $$r_{i}=\frac{c_{i}}{c_{0}},\;i\in [0,k]$$

(10) $$R=\left(\begin{array}{cccc} 1 & r_{1} & \cdots & r_{k-1}\\ \vdots & \vdots & \ddots & \vdots \\ r_{k-1} & r_{k-3} & \cdots & r_{1}\\ r_{k-1} & r_{k-1} & \cdots & 1 \end{array}\right)$$

(11) $$r=\left(\begin{array}{c} r_{1}\\ r_{2}\\ \vdots \\ r_{k-1}\\ r_{k} \end{array}\right)$$

The *ACF* and *PACF* of the model are calculated by choosing the appropriate autocorrelation order *p* and moving average order *q*. When the *ACF* converges quickly to 0 after being greater than a certain constant, this constant is called the autoregressive order *p*. Similarly, the *PACF* converges quickly after being greater than a certain constant, which is the moving average order *q*; then the model is fitted.

There is subjectivity in selecting *p* and *q* using *ACF* and *PACF*, so the Akaike information criterion (AIC) and Bayesian information criterion (BIC) are introduced to choose *p* and *q* more objectively, whose *AIC* can be expressed by Equation (12) and *BIC* can be described by Equation (13).

(12) AIC=−2ln(L)+2K

(13) BIC=−2ln(L)+Kln(n)

where *L* denotes the great likelihood function of the model, *K* denotes the number of model parameters, and *AIC* and *BIC* can balance the prediction error and model complexity, and according to the information criterion function, determine the model’s order.

After determining the difference order *d*, the autoregressive order *p* and the moving average order *q*, the parameters of the ARMIA(*p*, *d*, *q*) model are formed, based on which the input smoothed posterior time series are predicted as shown in the following equation.

(14) $$x_{t}=\alpha_{1}x_{t-1}+\alpha_{2}x_{t-2}+\cdots +\alpha_{p}x_{t-p}+u_{t}$$

where *x_t_* is the predicted output of the autoregressive model at moment *t*, *x_t_*,*i* ∈ [*t* − 1,*t − p*] is the historical input sequence, and *u_t_* is the random perturbation. When the random perturbation term is assumed to be white noise, Equation (14) can be rewritten in the following form.

(15) $$x_{t}=\alpha_{1}x_{t-1}+\alpha_{2}x_{t-2}+\cdots +\alpha_{p}x_{t-p}+\varepsilon_{t}$$

where *ε_t_* is the variance of white noise. *α_p_* is the weighting factor of the historical input series.

If *u_t_* is not white noise, a moving average of order *q* is used to represent it. That is:

(16) $$u_{t}=\varepsilon_{t}+\beta_{1}\varepsilon_{t-1}+\beta_{2}\varepsilon_{t-2}+\cdots +\beta_{q}\varepsilon_{t-q}$$

where *ε_t_*, *i* ∈ [*t* − 1,*t* − *q*] denotes the white noise series. *β_q_* is the weighting factor of the white noise sequence. In particular, when *x_t_ = u_t_*, i.e., the current value of the time series is not related to the historical values and depends only on the linear combination of historical white noise, the moving average term is obtained as:

(17) $$x_{t}=\varepsilon_{t}+\beta_{1}\varepsilon_{t-1}+\beta_{2}\varepsilon_{t-2}+\cdots +\beta_{q}\varepsilon_{t-q}$$

The ARIMA(*p*, *q*) is obtained by combining the autoregressive model and the moving average model based on the difference method, as shown in the following equation.

(18) $$x_{t}=\sum_{i=1}^{p}\alpha_{i}x_{t-i}+u_{t}+\sum_{j=1}^{q}\beta_{j}\varepsilon_{t-j}$$

### 3.2. Source–Load Scene Generation Based on Error Gradient Sampling Method

In order to generate specific source–load scenarios for the next 10 years, this paper will use the random sampling method to construct source–load scenarios for the next 10 years. However, in the random sampling process the source–load value at the future *t +* 1 moment will be constrained by the source–load value at the *t* moment, i.e., after determining the source–load scenario at the t moment, the source–load distribution at the *t +* 1 moment is not in satisfying the initial predicted distribution, to solve this problem this paper introduces the concept of source–load growth rate, whose specific expression is shown in Equations (19) and (20).

(19) $$r_{P}^{t}=\frac{P_{t+1}-P_{t}}{P_{t}}$$

(20) $$r_{P,\min}\leq r_{P}^{t}\leq r_{P,\max}$$

where *P_t+_*_1_ and *P_t_* are the source–load values at moments *t +* 1 and *t*, respectively, and *r_P_*_,min_ and *r_P_*_,max_ are the minimum and maximum growth rates of historical source–loads, respectively. The predicted distribution at the moment *t +* 1 is dynamically corrected in real time after randomly generating the source–load scenario at moment *t*, and the specific correction formula is as follows.

(21) $$P_{\min}^{t+1}=\left\{\begin{array}{l} P_{\mathrm{init},\min}^{t+1}\;\;\;\;\;\;\;\;\;\;\mathrm{if}\;P_{\mathrm{mean}}^{t+1}-P_{\min}^{t+1}>r_{P,\min}\\ P_{\mathrm{mean}}^{t+1}-r_{P,\min}\;\;\;\mathrm{else} \end{array}\right.\;\;\;\;\;$$

(22) $$P_{\max}^{t+1}=\left\{\begin{array}{l} P_{\mathrm{init},\max}^{t+1}\;\;\;\;\;\;\;\;\;\;\mathrm{if}\;P_{\max}^{t+1}-P_{\mathrm{mean}}^{t+1}>r_{P,\max}\\ P_{\mathrm{mean}}^{t+1}+r_{P,\max}\;\;\;\mathrm{else} \end{array}\right.\;\;\;\;\;$$

where *P_t+_*_1,max_, *P_t+_*_1,min_ and *P_t+_*_1,mean_ are the initial predicted maximum, minimum and trend baseline values of the source–load at time *t +* 1, respectively.

## 4. Multi-Stage Morphological Evolution Planning Model of Distribution Network

According to the above source–load prediction results, the distribution network can access a variety of flexible adjustment resources at different time points and adjust the operation mode of the network by optimizing its own scheduling or adding flexible adjustment resources. In this chapter, the multi-stage planning model of the distribution network is constructed to analyze the morphological evolution route of distribution network at different time points.

### 4.1. Objective Function

As the proportion of new energy sources such as wind and solar energy is gradually increasing, and demand-side resources are more widely involved in power grid operation and dispatching, when analyzing the evolution of distribution network, it is necessary to consider the development trend of customer-side resources and wind and solar resources. Based on this, the planning model of distribution network is constructed. The planning model takes the minimum planning cost of the distribution network system as the objective function, and considers the distribution network loss, system equipment investment cost and equipment operation cost, as shown in Equation (23).

(23) $$\min F_{1}=C_{S}+C_{M}+C_{e}E_{\mathrm{eloss}}+C_{Q}$$

(24) $$C_{S}=\sum_{j=1}^{N}\frac{C_{j}Q_{j}}{{\left(1+i\right)}^{n}}(1-t_{r})$$

(25) $$C_{M}=\sum_{n=1}^{L_{P}}\frac{C_{\mathrm{MP}}}{{\left(1+i\right)}^{n}}(1-t_{r})\alpha_{n}$$

(26) $$C_{\mathrm{MP}}=r_{M}\sum_{j=1}^{N}C_{j}Q_{j}$$

(27) $$C_{Q}=\sum_{n=1}^{L_{P}}\frac{C_{\mathrm{QP}}}{{\left(1-i\right)}^{n}}(1-t_{r})$$

(28) $$C_{\mathrm{QP}}=C_{r}P_{r}$$

where *C*_e_ denotes the time coefficient of power grid loss cost, *E*_eloss_ denotes the power loss of distribution network, *C*_s_ denotes the initial operation cost of power distribution system, *C*_M_ denotes the total cost of operation and maintenance of distribution system equipment, *C*_MP_ is the operation and maintenance cost of the equipment in the first year; *n* is the total number of equipment that the system needs to build; *C_j_* is the initial investment cost per unit capacity of equipment *j*; *Q_j_* is the capacity of equipment *j*; *t_r_* is the tax rate; *i* is the interest rate; *L_p_* is the equipment planning year; *r_M_* is the maintenance rate of the equipment; *α_n_* is the development degree of the system in the nth year; *C*_Q_ is the cost of abandoned wind and light, *C*_QP_ is the loss of abandoned wind and light in the first year of the system; *C*_r_ is the penalty coefficient of abandoned wind and light, and *P*_r_ is the amount of abandoned wind and light.

### 4.2. Constraint Condition

#### 4.2.1. Equipment Capacity Constraint

The capacity constraint of the above involved devices is shown in Equation (29).

(29) $$\left\{\begin{array}{c} Q_{\mathrm{EX},k}^{\min}\leq Q_{\mathrm{EX},k}\leq Q_{\mathrm{EX},k}^{\max}\\ P_{j}^{\min}\leq P_{j}\leq P_{j}^{\max}\\ P_{u,j}^{\min}\leq P_{u,j}\leq P_{u,j}^{\max}\\ P_{d,j}^{\min}\leq P_{d,j}\leq P_{d,j}^{\max} \end{array}\right.$$

where *Q*_EX,*k*_ denotes the actual reserve capacity of the resource *k*, $Q_{\mathrm{EX},k}^{\min}$ and $Q_{\mathrm{EX},k}^{\max}$ denote the minimum and maximum reserve capacity of the resource *k*, *P_j_* denotes the actual output of the device *j*, $P_{j}^{\min}$ and $P_{j}^{\max}$ denote the minimum and maximum output of the device *j*, *P*_u__,*j*_ denotes the actual upward climbing rate of the device *j*, $P_{d,j}^{\min}$ and $P_{u,j}^{\max}$ denote the minimum and maximum upward climbing rate of the device *j*, *P*_d__,*j*_ denotes the actual downward climbing rate of the device *j*, $P_{d,j}^{min}$ and $P_{d,j}^{max}$ denote the minimum and maximum downward climbing rate of the device *j*.

#### 4.2.2. Power Grid Operation Constraint

The distribution network system model mainly includes power balance constraint, nodal voltage constraint, three-phase power flow constraint, line power constraint, generator output constraint, and generator climbing constraint.

(30) $$\sum_{j\in \Omega_{l}^{i}}P_{ij,t}=\sum_{g\in \Omega_{G}^{i}}P_{g,t}+P_{i,t}^{WT}+P_{i,t}^{PV}-P_{i,t}^{\mathrm{indl}}-P_{i,t}^{\mathrm{ecol}}-P_{i,t}^{\mathrm{resl}}+P_{i,t}^{EV,in}-P_{i,t}^{EV,out}$$

(31) $$\sum_{j\in \Omega_{l}^{i}}Q_{ij,t}=\sum_{g\in \Omega_{G}^{i}}Q_{g,t}-Q_{i,t}^{EV}-Q_{i,t}^{\mathrm{indl}}-Q_{i,t}^{\mathrm{ecol}}-Q_{i,t}^{\mathrm{resl}}$$

(32) $$V_{i}^{\min}\leq V_{i,t}\leq V_{i}^{\max}$$

(33) $$\sum_{j\in \Omega_{l}^{i}}P_{ij,t}-V_{i,t}\sum_{j\in \Omega_{l}^{i}}V_{j,t}\left(G_{ij}\cos\theta_{ij}+B_{ij}\sin\theta_{ij}\right)=0$$

(34) $$\sum_{j\in \Omega_{l}^{i}}Q_{ij,t}-V_{i,t}\sum_{j\in \Omega_{l}^{i}}V_{j,t}\left(G_{ij}\sin\theta_{ij}-B_{ij}\cos\theta_{ij}\right)=0$$

(35) $$-P_{ij}^{\max}\leq P_{ij,t}\leq P_{ij}^{\max}$$

(36) $$P_{g}^{\min}\leq P_{g,t}\leq P_{g}^{\max}$$

(37) $$Q_{g}^{\min}\leq Q_{g,t}\leq Q_{g}^{\max}$$

(38) $$P_{g,t}-P_{g,t-1}\leq RU_{g}$$

(39) $$P_{g,t-1}-P_{g,t}\leq RD_{g}$$

where *P_ij_*_,*t*_ denotes the transmission power between node *I* and node *j* at time *t*, *P_g_*_,*t*_ denotes the active power of the generator at time *t*, $P_{i,t}^{WT}$ denotes the active power of wind power at node *i* at time *t*, $P_{i,t}^{PV}$ denotes the active power of photovoltaic at node *i* at time *t*, $P_{i,t}^{indl}$ denotes the industrial load power at node *i*, $P_{i,t}^{\mathrm{ecol}}$ denotes the commercial load power at node *i*, $P_{i,t}^{\mathrm{resl}}$ denotes the residential load power at node *i*, $P_{i,t}^{EV,in}$ and $P_{i,t}^{EV,out}$ denote the charging power and the discharge power of the electric vehicle at node *i* at time *t*, *Q_ij_*_,*t*_ denotes the reactive power between node *i* and node *j* at time *t*, *Q_g_*_,*t*_ denotes the reactive power of the generator at time *t*, $Q_{i,t}^{EV}$ denotes the reactive power of electric vehicle of node *i* at time *t*, $Q_{i,t}^{\mathrm{indl}}$ denotes the industrial load reactive power at node *i*, $Q_{i,t}^{\mathrm{ecol}}$ denotes the commercial load reactive power at node *i*, $Q_{i,t}^{\mathrm{resl}}$ denotes the residential load reactive power at node *i*, *V_i_*_,*t*_ denotes the voltage of node *i* at time *t*, *G_ij_* denotes the conductance between node *i* and node *j* at time *t*, *B_ij_* denotes the susceptance between node *i* and node *j*, *θ_ij_* denotes the voltage phase angle between node *i* and node *j*, $P_{ij}^{\max}$ denotes the maximum transmission power, $P_{g}^{\min}$, $P_{g}^{\max}$ denote the upper and lower limits of the generator active power, respectively, $Q_{g}^{\min}$, $Q_{g}^{\max}$ denote the upper and lower limits of the generator reactive power, respectively, *RU_g_*, *RD_g_* denote the upper and lower climbing limits of the generator.

## 5. Morphological Evolution Planning Analysis of Typical Distribution Network Based on Source–Load Prediction

**Figure 2:**
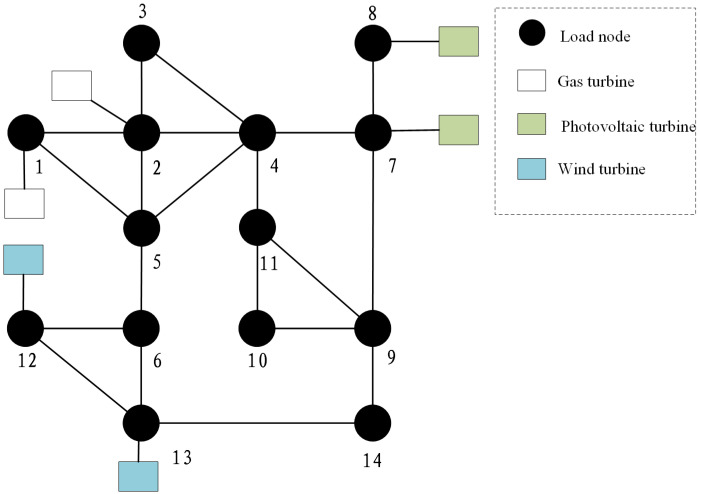
IEEE14 node distribution system.

In this paper, the source–load prediction model proposed in Section 4.1 is used to predict the trend of the nodes of the typical example system. The example system is shown in Figure 2.

In this example, the load demand of each node is equivalently converted from the predicted load of typical areas. Among them, nodes 1, node 5, node 6, node 7, and node 10 are mainly industrial loads, and the total load of nodes 1 and 7 is higher among all nodes. The total load of nodes 2, 8, and 9 is lower, the economic growth of the region represented by node 2 is slower, and the commercial and industrial loads account for a more average share. Nodes 8 and node 9 are relatively small and dominated by commercial loads due to their geographical location; load distribution and the total amount of remaining nodes are more uniform.

Nodes 1, 4, and 7 also have a higher demand for electric vehicles, which is consistent with their larger industrial loads. Due to the impact of the epidemic, the load growth trend of each node is small before 2024, the economy is on track after 2024, and the population of residents in various regions is gradually rising, driving the load growth of the commercial complex. With the morphological evolution of the distribution network, some nodes will be connected to new energy, and urban users will also carry out some demand response behavior of adjustable load.

In order to verify the effectiveness of the method proposed in this paper, two scenarios are set up for comparative analysis:

S1: Single-stage planning scenario of distribution network considering the source–load prediction;

S2: Multi-stage planning scenario of distribution network considering the source–load prediction.

The effectiveness of the proposed method is verified by comparing the results of the two scenarios.

### 5.1. Prediction Analysis of the Coming Source–Load Morphology in the Distribution Network

#### 5.1.1. Prediction Analysis of the Coming Distributed Generations Morphology in the Distribution Network

**Figure 3:**
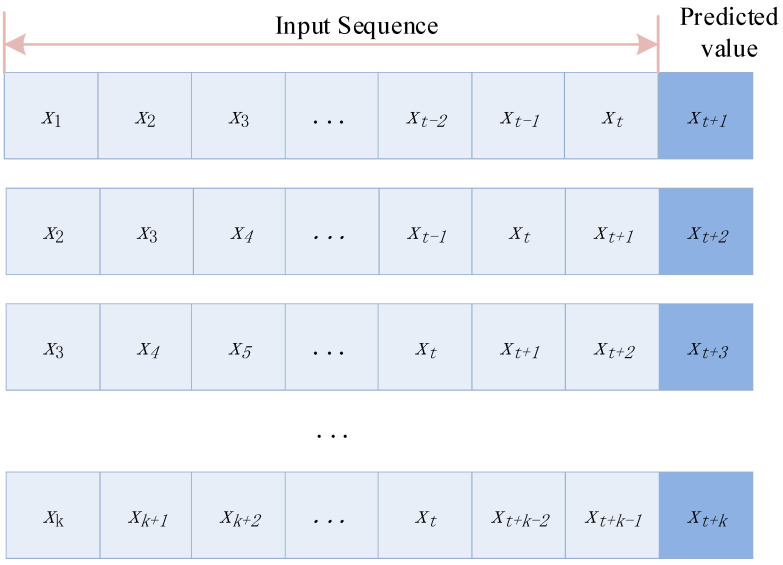
Rolling forecast diagram.

As the ARIMA model is not capable of multi-step forecasting, a rolling forecast is used for multi-step forecasting, and a schematic diagram of the rolling forecast is shown in Figure 3.

**Figure 4:**
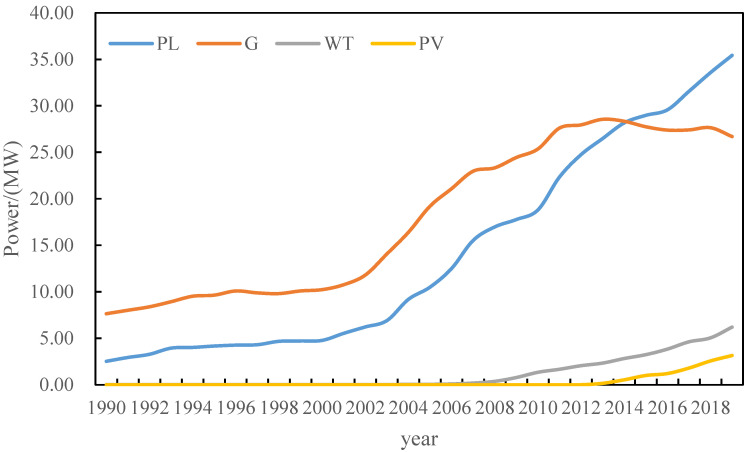
Model input historical data.

The annual load, gas turbine output, wind power output, and photovoltaic output data of a region are used to predict the annual load, gas turbine output, wind power output, and photovoltaic output of the region in the next 10 years. The data of load, gas turbine output, wind power output, and photovoltaic output over the years are shown in Figure 4.

In Figure 4, blue is the historical output curve of gas turbines. It can be found that the output of gas turbines shows a downward trend from 2013 to 2019. The red is the wind power output curve, and the wind power output is in a high growth state. The yellow is the photovoltaic output curve, and the photovoltaic output is small, but the overall trend is still growing.

**Figure 5:**
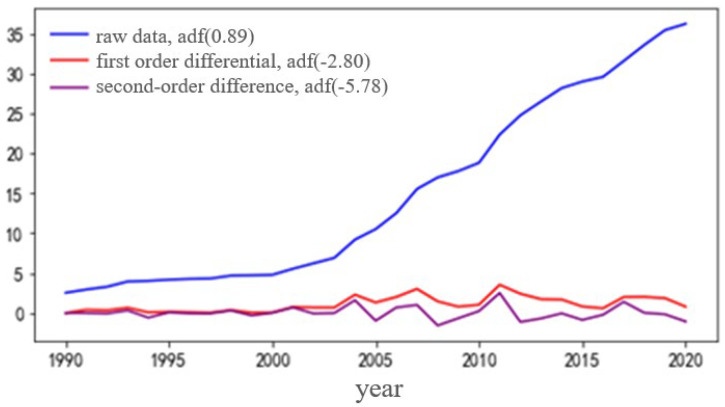
Load data stability detection.

Firstly, the ARIMA model is established for the historical load data, and the ADF unit root is used to test the stability of the original historical load, first-order difference, and second-order difference sequence. The results are shown in Figure 5.

It can be seen from Figure 5 that the *ADF* unit root of the second-order difference sequence is −5.78 much smaller than the *ACF* unit root of the first-order difference and the original sequence, indicating that the stability of the second-order difference is stronger, and it can be seen that the values fluctuate around 0, so the difference coefficient d of ARIMA is 2.

**Figure 6:**
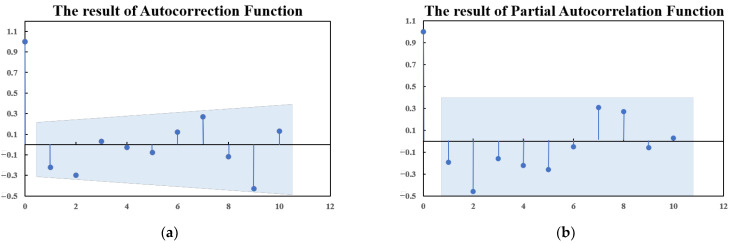
The results of *ACF* (**a**) and *PACF* (**b**).

*ACF* and *PACF* are used to select *p* and *q* for the ARIMA model. The results of *ACF* and *PACF* for the second-order differential load sequences are shown in Figure 6.

It can be seen from Figure 6 that both *ACF* and *PACF* are first-order truncations, that is the value of the sequence becomes very small after the first point, so *p* and *q* should be taken as 1. However, due to the subjectivity of using *ACF* and *PACF* to select *p* and *q*, *p* and *q* are corrected by Equations (12) and (13).

**Figure 7:**
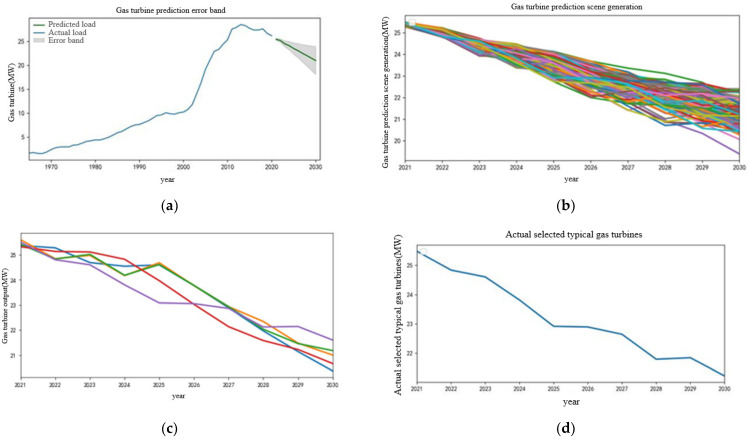
Gas turbine output prediction results. (**a**) Gas turbine prediction error band. (**b**) Gas turbine prediction scene generation. (**c**) Gas turbine output scenarios for the next 10 years after reduction. (**d**) Actual selected prediction curve of gas turbine.

**Figure 8:**
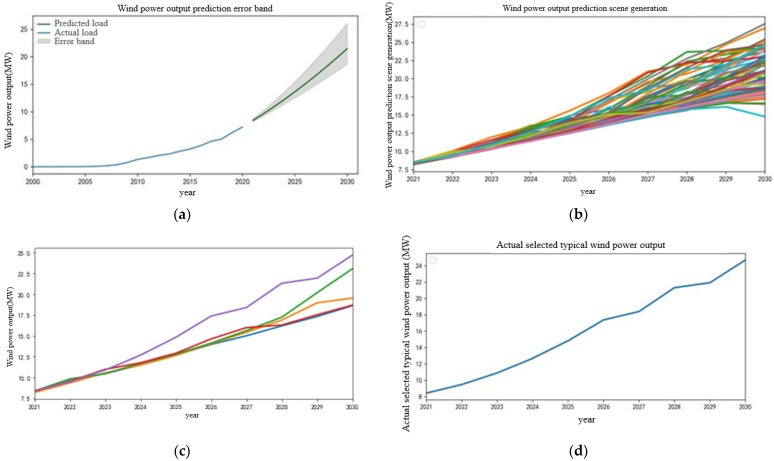
Wind power output forecast chart. (**a**) Wind power output prediction error band. (**b**) Wind power output prediction scenario generation. (**c**) Wind power output scenarios in the next 10 years after reduction. (**d**) Actual selected wind power output scenarios.

**Figure 9:**
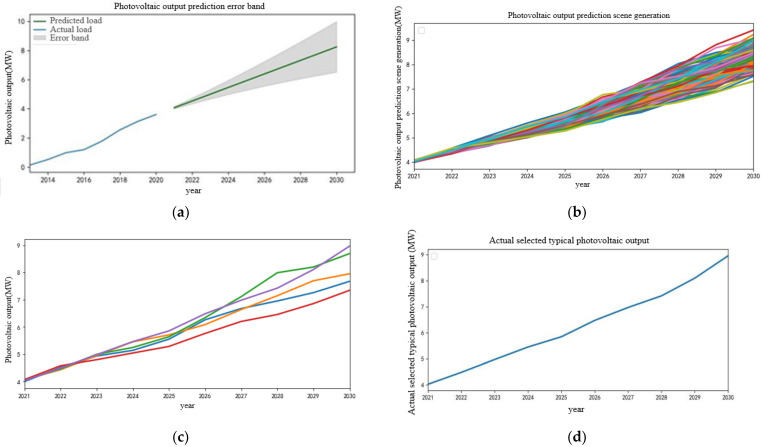
Photovoltaic output prediction results. (**a**) Photovoltaic output prediction error band. (**b**) Photovoltaic output prediction scenario generation. (**c**) Photovoltaic output scenarios for the next 10 years after reduction. (**d**) Actual selected photovoltaic output scenarios.

The prediction process of gas turbine output, wind power output, and photovoltaic output in the next 10 years is similar to the appeal process. The relevant process is no longer given specifically, and the modeling results and prediction results are given directly. The modeling results are ARIMA (1,2,3), ARIMA (1,2,0), ARIMA (0,2,0). The prediction results are shown in Figure 7, Figure 8 and Figure 9, respectively.

On the basis of the historical gas turbine output curve, combined with the characteristics of the distributed generation at the present stage [24], the prediction trend and error interval of the gas turbine in the next 10 years are formed. Due to the large-scale access to new energy in the future, the utilization rate of primary energy is gradually reduced, and the output of the traditional unit also shows a gradual downward trend. Because the future energy development trend cannot be accurately obtained, the rolling prediction is carried out by using the three times variance of the data of adjacent years, so the error interval will be generated, as shown in Figure 7a. The proposed error gradient sampling method is used to generate the scene of the gas turbine output trend in the error interval, and the K-means clustering is used to reduce the generated scene, as shown in Figure 7b,c. On this basis, the final output curve of the selected gas turbine in the next 10 years is shown in Figure 7d, and the overall output curve shows a downward trend.

There are abundant wind power resources in some areas of China. With the development of new power systems and the breakthrough of wind power technology, the output of wind power in China has generally shown an upward trend in the past 10 years. Based on the analysis of the development trend of wind power [25], ARIMA combined with the principle of three times variance is used to predict the output of wind power in the next 10 years, and the trend and error interval of wind power output in the next 10 years is obtained, as shown in Figure 8. On this basis, the error gradient sampling method is used to generate multiple wind power output trend scenarios in the error interval and K-means clustering is used to reduce them, as shown in Figure 8b,c. The final selected wind power output scenario is shown in Figure 8d. It can be seen that the wind power output will show a rapid upward trend in the next 10 years, but the update in technology can drive the development of wind power resources. Therefore, the growth trend of wind power output will occasionally slow down in the next 10 years.

From the historical data of photovoltaic, it can be found that its historical output curve is similar to that of wind power. ARIMA and three times variance principle are used to predict the photovoltaic output, and the output curve and error interval of photovoltaic in the next 10 years are obtained, as shown in Figure 9a. The error gradient sampling method is used to generate the photovoltaic output scene in the error interval, and the K-means clustering is used to reduce the scene. Finally, the typical photovoltaic output scene is selected, as shown in Figure 9b,d. It can be seen from the diagram that photovoltaic output is similar to wind power output, and it is also growing rapidly in the next 10 years, which is in line with the characteristics of China’s future new power system construction. In the future, wind power and photovoltaics will be connected to the power grid on a large scale, gradually occupying the main body of power generation in China.

#### 5.1.2. Prediction Analysis of the Coming Multi-Type Load Morphology in the Distribution Network

**Figure 10:**
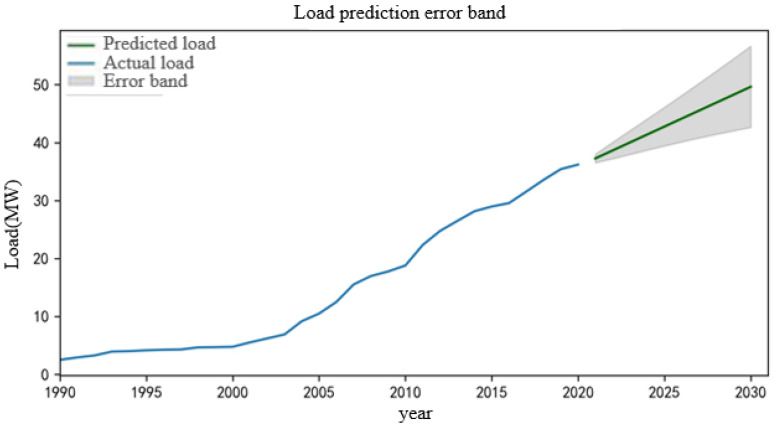
Initial load prediction results.

The network search method is used to search and traverse between *p* ∈ [0, 5] and *q* ∈ [0, 5]. Finally, the minimum AIC is 72.227 when *p* = 0, *q* = 2, so the ARIMA (0, 2, 2) model is finally established. The model is trained, and the load data for the next 10 years are predicted. The results are shown in Figure 10.

**Figure 11:**
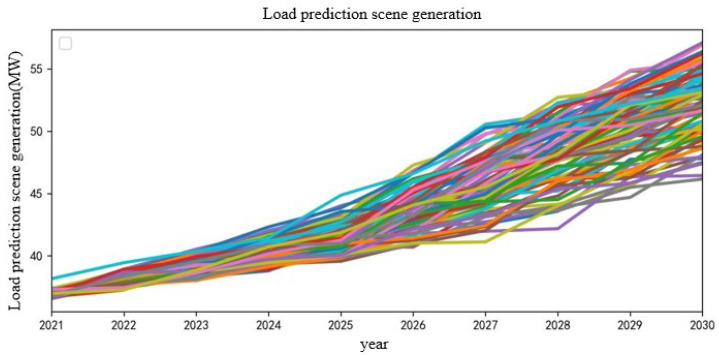
Final load prediction results.

From Figure 10, it can be seen that the load in the region will show an increasing trend in the next 10 years, and its value fluctuates within the error range that satisfies the Gaussian distribution. After processing by this method, the final load scenario of node 1 in the next 10 years is shown in Figure 11.

**Figure 12:**
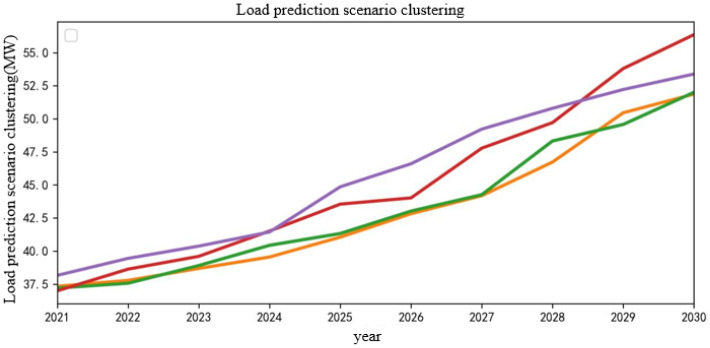
Load scenarios in the next 10 years after reduction.

**Figure 13:**
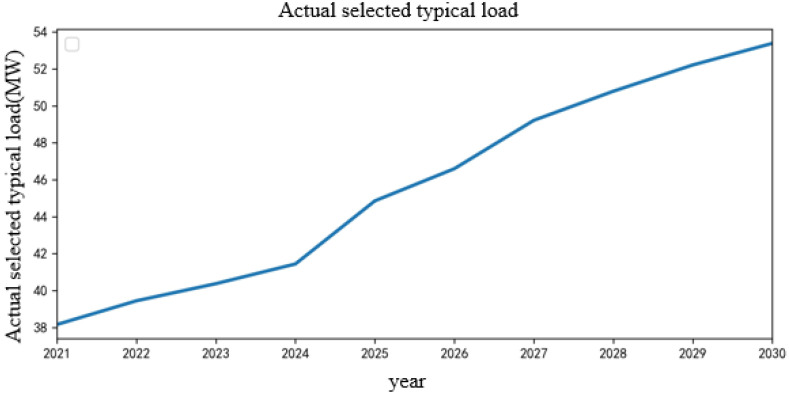
Actual selected typical load scenarios.

Then, K-means clustering is used to reduce the generated scenarios, and the typical load scenarios in the next 10 years are obtained. The clustering center *k* is 4, and the typical load scenarios in the next 10 years after reductions are obtained as shown in Figure 12. According to the obtained clustering results, a typical scene is randomly selected for analysis, and the typical scene is selected as shown in Figure 13.

**Figure 14:**
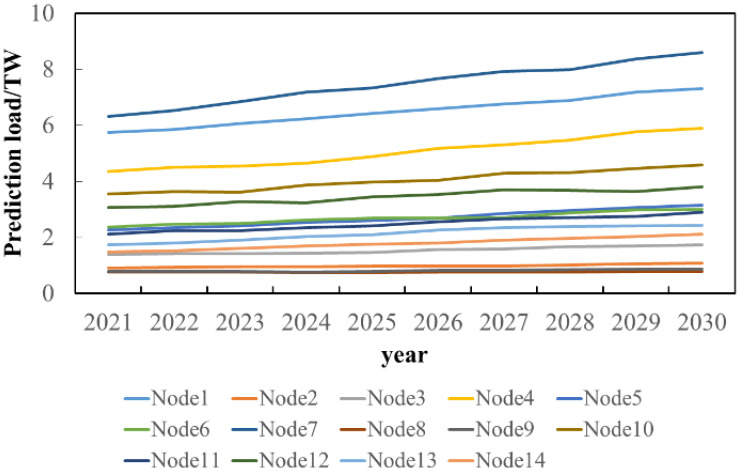
Load prediction results of each node.

Using the same method, the load scenarios of other nodes in the next 10 years are obtained, and the results are shown in Figure 14.

**Figure 15:**
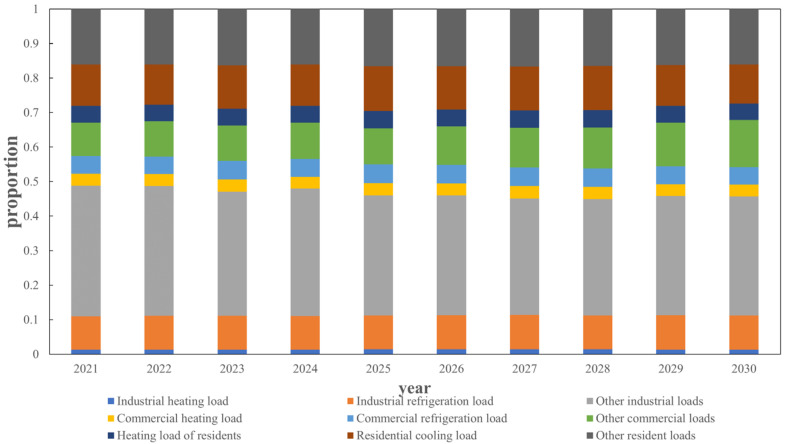
Node 1 load proportion prediction.

**Figure 16:**
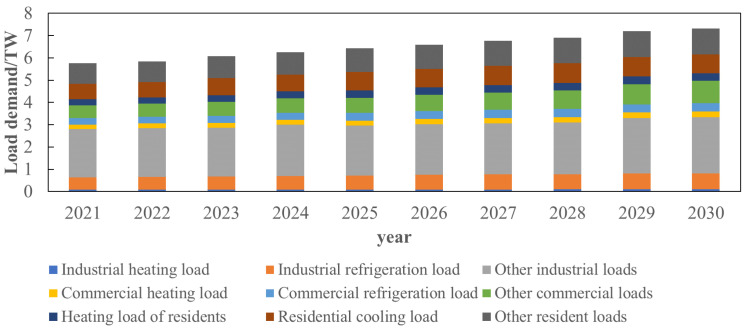
Node 1 load prediction by type.

It can be seen from Figure 14 that the load of each node is on the rise, with nodes 1, 4, and 7 showing a large increase and the other nodes showing a slow growth trend in load. At the same time, in order to study the composition of each node load, the proportion of industrial heating load, industrial refrigeration load, other industrial load, commercial heating load, commercial refrigeration load, other commercial load, residential heating load, residential refrigeration load and other residential load of each node from 2010 to 2020 is analyzed, and the proportion distribution of each node load in the next 10 years is analyzed by statistical method. For the load with obvious change trend, the mean and variance of its growth rate are calculated, and the growth rate at time *t* + 1 is randomly selected by using the 3σ principle. For the load with no obvious change trend, the mean and variance of its proportion are counted, and the proportion at *t* + 1 is randomly selected by using the 3σ principle. The specific formula is as follows:

(40) $$P_{o}^{t+1}=P_{o}^{t}+\mathrm{random}(r_{P,\mathrm{mean}},r_{P,\mathrm{std}})P_{o}^{t}$$

(41) $$P_{\mathrm{no}}^{t+1}=\mathrm{random}(P_{\mathrm{no},\mathrm{mean}},P_{\mathrm{no},\mathrm{std}})$$

where $p_{o}^{t+1}$, $p_{o}^{t}$ denote the load values with obvious proportion changes at tome *t* + 1 and *t*, $p_{no}^{t+1}$ denotes the load value whose proportion change is not obvious at time *t* + 1, *r_P_*_,mean_ and *r_P_*_,std_ denote the mean and variance of the obvious load growth rate of the proportion change, *P*_no,mean_ and *P*_no,std_ denote the mean and variance of the load with no obvious change in the proportion, and random (*) is a random function. The proportion of each type of load at node 1 from 2021 to 2030 is obtained through the appeal method, as shown in Figure 15 and Figure 16.

As shown in Figure 15, the load of node 1 in the next 10 years is mainly industrial load, accounting for about 50% of the total load demand. Among them, the proportion of other industrial loads is about 0.35, and the proportion of commercial load and residential load is about 0.25. The proportion of load in node 1 in 10 years is basically unchanged, and the proportion of industrial load has declined. On the contrary, the proportion of commercial load has increased. This is mainly due to the regional load is always the main part of the industrial load, large industrial users occupy a major position. It can be seen from Figure 16 that although the change in proportion of load is small, the annual load demand continues to increase. The total load of node 1 in 2030 is about 27.09% higher than that in 2021, which is mainly due to China ‘s future economic development and population. From 2021 to 2030, the industrial load will increase by about 19.06%, the commercial load will increase by about 53.89%, and the residential load will increase by about 24.12%. It can be seen that the commercial load will have a greater growth trend in the next 10 years, mainly because the development of the city will eventually be biased towards comfort and user satisfaction. The industrial load will gradually shift to areas with less cost or population, and the commercial load in areas with more population will gradually increase. The industrial, commercial and residential loads only account for a small proportion of the hot and cold loads, and the loads are still dominated by other loads.

### 5.2. Analysis of Multi-Stage Configuration Results

#### 5.2.1. Multi-Stage Planning Results of Distribution Network

**Figure 17:**
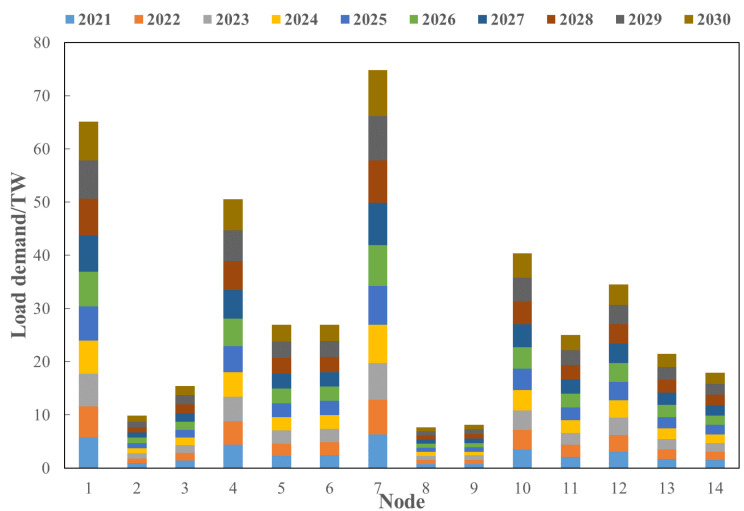
Load demand by node from 2020 to 2030.

Aiming at the source–load demand from 2021 to 2030 predicted in Section 3.2, based on the IEEE 14-node distribution system, the evolution route of the distribution network in the next ten years is analyzed under the predicted typical source–load scenarios. The node source–load data of the 14-node distribution system is shown in Figure 17.

**Figure 18:**
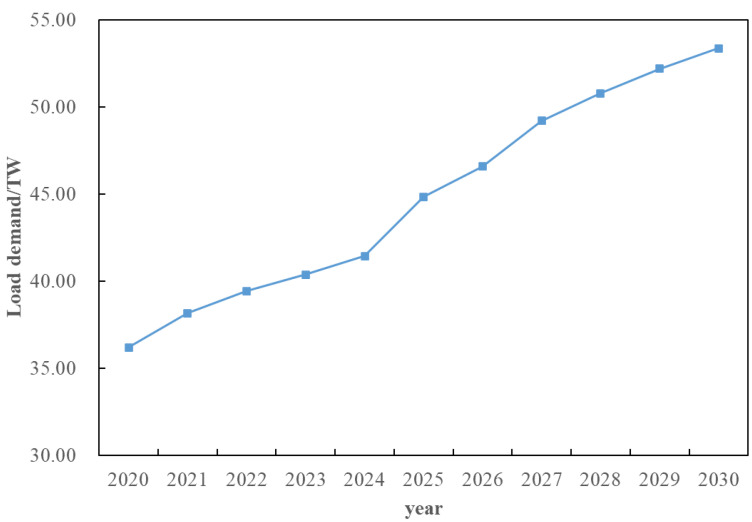
Load growth trend from 2020 to 2030.

**Figure 19:**
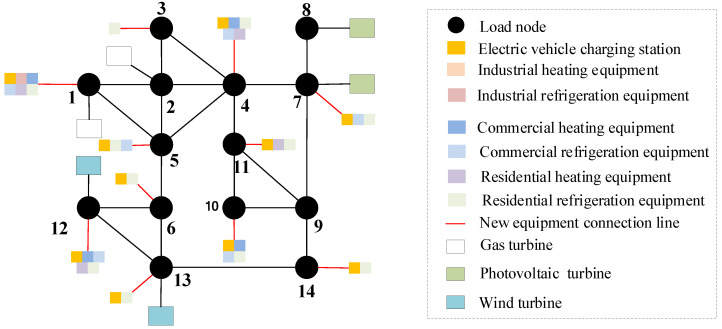
Morphological evolution of distribution network in 2021.

**Figure 20:**
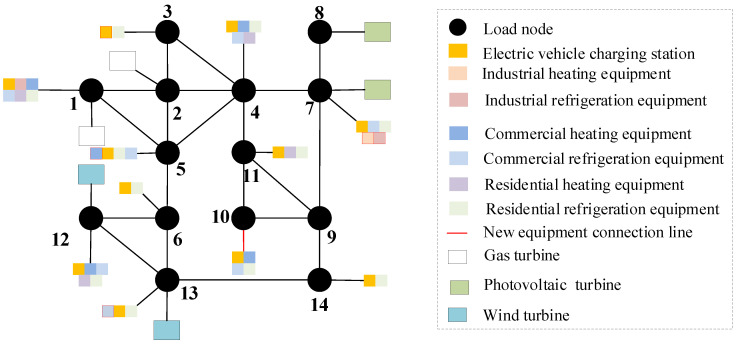
Morphological evolution of distribution network in 2024.

**Figure 21:**
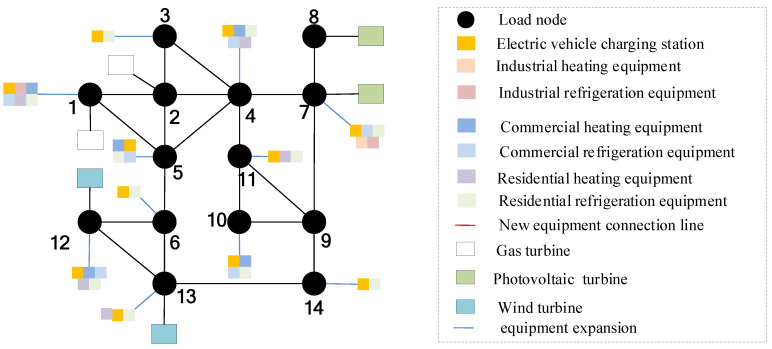
Morphological evolution of distribution network in 2025.

**Figure 22:**
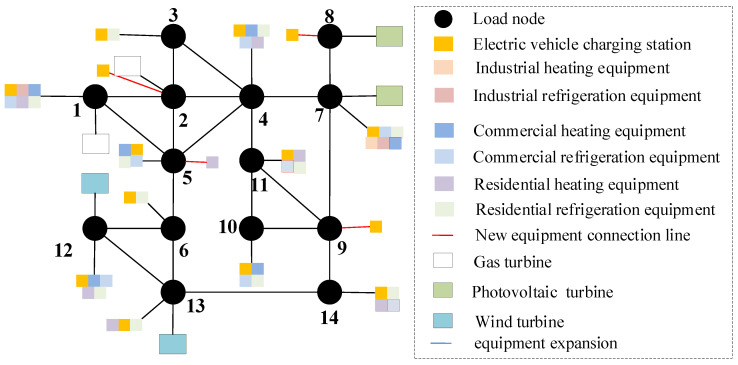
Morphological evolution of distribution network in 2028.

On the basis of Section 5.3, consider the time cross-section of source–load development, as shown in Figure 18. Combined with the source–load prediction results and development trend in Section 5.2, it can be seen that in 2025, the load demand shows a large increase, compared with the previous year increased by about 25%, and by 2030, the total load demand increased by 40%. Therefore, considering that there is a time cross-section from 2021 to 2030, a multi-stage evolution route of the distribution network is formed. As shown in Table 1 and Table 2. The morphological evolution of distribution network is shown in Figure 19, Figure 20, Figure 21 and Figure 22.

As a result of the double carbon target, China has started to reduce carbon emissions, and with cars as the main greenhouse gas emitters, it is an inevitable trend to study the use of clean energy electric vehicles. Therefore, in the future evolution of the distribution network, electric vehicles will participate in power grid planning as the main load. By comparing Figure 19, Figure 20, Figure 21 and Figure 22, considering the load growth cross-section in 2025, it can be found that the increase in electric vehicles, industrial and commercial refrigeration, and heating equipment shows a certain timeliness, mostly concentrated in 2021 and 2023. This is because before 2025, the load change trend is small, and the electric vehicles, industrial, commercial and residential refrigeration and heating equipment configured in 2021 can effectively meet the overall operation of the distribution network. The power grid can rely on its own dispatching operation to ensure the balance of load supply and demand, and there is no need to configure new equipment. In 2025, as the load shows massive growth and the scale of new energy is expanding before 2025 and new energy is only considered for local consumption, but after 2025, the installed capacity of new energy increases massively, and the original local balance has been unable to meet the consumption of new energy such as wind power and photovoltaic. On the basis of adding electric vehicles, and industrial, commercial, and residential refrigeration and heating equipment, new energy gradually forms cross-regional return with external power grids to promote the consumption of new energy. It can be seen from Table 2 that in the distribution network planning from 2025 to 2030, the configuration scheme of the electric vehicle charging station of node 8 and node 9 is added, and the residential heating equipment is added at nodes 5 and 14.

#### 5.2.2. Comparative Analysis of Different Scene Configuration Results

Based on the above-proposed distribution network source–load prediction results, the distribution network is planned in a single phase without considering the increase or decrease in source–load in a specific year. The development of the distribution network is always accompanied by changes in energy, the alternation of new and old towns, and the addition of emerging entities (such as factories, load aggregators, residential communities, etc.). In view of the changes in node load demand and the trend of total node load under the access of different entities, considering the configuration of electric vehicles, industrial, commercial, and residential heating equipment and refrigeration equipment, the results of resource optimization configuration of the distribution network from 2021 to 2030 are shown in Table 3.

Through the comparison of planning costs in the S1 and S2 scenarios, it can be found that the total planning cost in the S1 scenario is about CNY 121,900.69, and the planning cost in the S2 scenario is CNY 117,289.49. Compared with the S1 scenario, the S2 planning cost considering the multi-stage planning scenario is reduced by about 3%, indicating that multi-stage planning can better improve the economy of planning compared with single-stage planning. From Table 3, it can be found that when the single-stage planning is carried out without considering the change in the load cross-section, compared with the configuration results of S2, most of the equipment in the S1 scenario is mainly configured in the first year of planning, and the configuration capacity is large. This is mainly because in the single-stage planning, the time cross-section change is not considered, and the cost of the entire planning cycle is minimized. The majority of the capacity allocation in the first year of planning allows for optimal system planning costs. Electric vehicle charging stations are planned at each node, and mostly in the first year. According to the load prediction results of 5.1, it can be seen that the electric vehicle load of each node of the system shows an increasing trend year by year, while the electric vehicle load of node 1 and node 7 accounts for a large proportion of the electric vehicle load of the whole system. Calculating the proportion of electric vehicles to the total node load for all nodes shows that there is less variability in the proportion of electric vehicles at each node, although node 1 and node 7 have a much larger electric vehicles allocation capacity than the other nodes, this is also a result of the total load at node 1 and node 7 having a larger demand relative to the other nodes, and therefore nodes 1 and 7 have a much larger electric vehicles charging station allocation capacity than the other nodes.

The configuration of industrial equipment is mostly concentrated on node 1 and node 7, which is mainly due to the large proportion of industrial load of these two nodes, and the relative proportion of industrial heat load demand of node 7 is relatively large. However, in the early stage of planning, the thermal load demand of nodes can be met through the dispatching instructions of power grid, so the industrial heating equipment will not be configured until 2023. For industrial refrigeration, the cooling load demand of node 1 is high, and the refrigeration equipment configuration has been carried out in 2021. When configuring equipment for commercial loads, nodes 3, 8, and 9 are considered to have a small geographical area and a smaller overall share of the load required relative to the other nodes, while nodes 4 and 5 have a larger demand, so these two nodes are allocated a large proportion of the capacity.

Comparing the configuration results of the S1 and S2 scenarios, namely Table 2 and Table 3, it can be found that compared with the single-stage planning results, when considering 2025 as the time cross-section, the distribution network does not need to plan most of the capacity in the first year, which can effectively reduce the cost of the equipment configuration. Before the second stage configuration, the system can balance the source–load through its own optimal scheduling. When the line reaches the maximum capacity or the load demand increases significantly, the second configuration is performed. Compared with the overall configuration of the single-stage configuration in the first year, the equipment configuration considering the time cross-section of the distribution network can effectively reduce the planning cost of the distribution network and flexibly adjust the planning scheme of the power grid.

### 5.3. Analysis of Distribution Network Dispatch Results during No New Equipment

**Figure 23:**
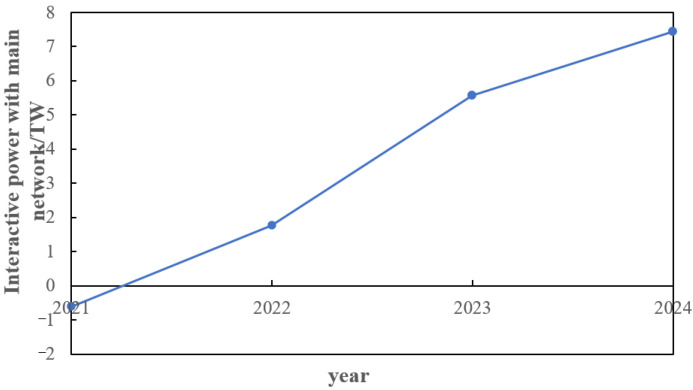
Interactive power between distribution network and main network from 2021 to 2024 years.

From the above analysis, it can be found that the distribution network equipment is only configured in a certain year, and in the unconfigured year, the power grid needs to reasonably plan the power output through its own dispatching operation to achieve the balance of supply and demand of each node in the network. The power output curve of the interaction between the distribution network and the external power grid in each stage year is shown in Figure 23.

**Figure 24:**
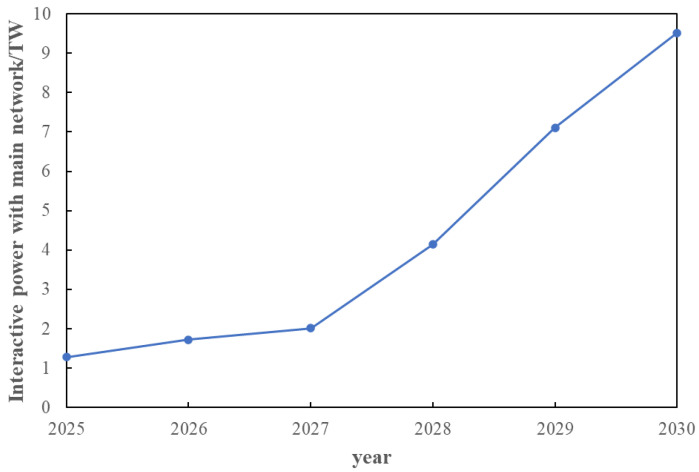
Interactive power between distribution network and main network from 2025 to 2030 years.

It can be seen from the Figure 23 that after the capacity configuration of the distribution network is completed in 2021 because the load is far less than the configuration capacity, the new energy unit has additional output to help the main network to consume; after that, due to the continuous growth of the load, the power output in the distribution network is not enough to meet the load demand. At this time, it is necessary to purchase electricity from the main network to provide the node load, so the interactive power shows an increase. In 2025 form Figure 24, due to the new addition and expansion of node equipment in the distribution network, the pressure on new energy output is alleviated, and the interactive power is reduced compared with 2024, but it still shows an overall growth trend.

**Figure 25:**
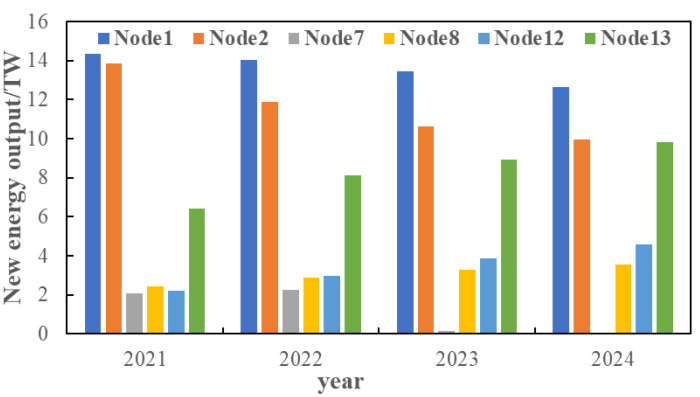
Power output of each power supply in the distribution network from 2020 to 2024.

Between 2020 and 2024, the power output of the distribution network is shown in Figure 25.

It can be seen from the above that 2024 is a time cross-section of load change. Before 2024, the load growth trend is relatively flat. According to Table 1, compared with the single-stage planning evolution, the equipment configuration capacity is smaller. Therefore, the power output is dominated by the gas turbines at node 1 and node 2, with a smaller output from the new energy units, and the output of each unit can meet the load demand at each node of the distribution network.

**Figure 26:**
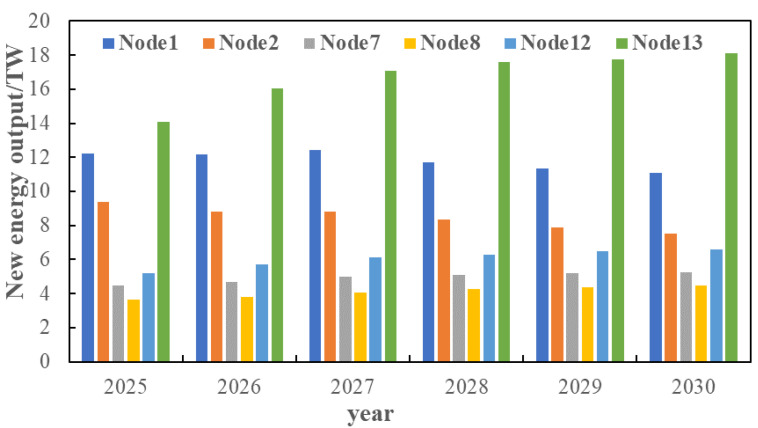
Power output of each power supply in the distribution network from 2025 to 2030.

It can be seen from Figure 26 that the load will increase greatly in 2025, which is the result of the economic recovery after the domestic epidemic and the construction of new towns. Therefore, most of the equipment expansion or new equipment is carried out in 2025 to meet the demand of power grid load. At the same time, it can be seen from Figure 26 that traditional units are gradually replaced by new energy units, and new energy units gradually become the main power supply on the supply side. The power supply in each year is combined by the units to create an overall output to meet the load variations of the distribution network.

## 6. Conclusions

In this paper, ARIMA and error gradient sampling methods are used to predict the future power structure and load demand of the distribution network, and then the multi-stage planning of a typical distribution network is carried out based on the prediction results and planning model of the coming source–load morphology. The conclusions obtained are shown below.

1. The ARIMA prediction model can be used to effectively explore the trend of power supply and load and take into account the smoothness of the prediction sequence. The error gradient sampling method can effectively divide the error interval formed by the prediction. The combination of ARIMA and error gradient sampling method can effectively predict the trend of the power structure and load demand scale;
2. The proposed multi-stage planning model of a typical distribution network based on source–load prediction can realize the capacity planning of different types of equipment in different stages of the distribution network on the basis of reducing the planning cost. Compared with single-stage planning, it can effectively reduce the cost of equipment investment and adjust the planning scheme of the distribution network more flexibly;
3. This paper mainly considers the influence of changes in source–load development on the rigorous route of distribution network planning. With the development and innovation of new energy and load technology in the future and the change in electricity demand and resources of multi-type users, the future distribution network planning also needs to combine the factors of scientific and technological development and the driving conditions of resources and electricity demand, so as to form a comprehensive and flexible planning of distribution network.

## Figures and Tables

**Table 1:** Resource allocation results of distribution network from 2020 to 2025.

Resource Type	Node	Time/(Year)	Capacity/(MW)
Electric vehicle	1	2021	0.955
	3	2022	0.225
	4	2021	0.759
	5	2021	0.368
	6	2021	0.439
	7	2021	1.44
	10	2021	0.574
	11	2021	0.345
	12	2021	0.409
	13	2021	0.324
	14	2021	0.249
Industrial heating	7	2023	0.176
Industrial refrigeration	1	2021	0.724
	7	2023	0.169
Commercial heating	1	2021	0.234
	4	2021	0.234
	5	2023	0.155
	10	2021	0.169
	12	2021	0.183
Commercial refrigeration	1	2021	0.365
	4	2021	0.46
	5	2021	0.392
	7	2021	0.381
	10	2021	0.344
	12	2021	0.226
	13	2023	0.174
Residential heating	1	2021	0.339
	4	2021	0.285
	11	2021	0.343
	12	2021	0.268
Residential refrigeration	1	2021	0.921
	3	2021	0.166
	4	2021	0.56
	5	2021	0.445
	6	2021	0.326
	7	2021	0.716
	10	2021	0.346
	11	2021	0.533
	12	2021	0.674
	13	2021	0.311
	14	2021	0.25

**Table 2:** Optimal allocation results of distribution network resources from 2025 to 2030.

Resource Type	Node	Time/(Year)	Capacity/(MW)
Electric vehicle	1	2025	2.129
	2	2026	0.24
	3	2025	0.536
	4	2025	1.856
	5	2025	0.843
	6	2025	0.876
	7	2025	2.571
	8	2029	0.206
	9	2028	0.211
	10	2025	1.23
	11	2025	0.756
	12	2025	0.986
	13	2025	0.682
	14	2025	0.565
Industrial heating	7	2025	0.22
Industrial refrigeration	1	2025	0.866
	7	2025	0.209
Commercial heating	1	2025	0.279
	4	2025	0.306
	5	2025	0.206
	7	2027	0.179
	10	2025	0.211
	12	2025	0.22
Commercial refrigeration	1	2025	0.426
	4	2025	0.591
	5	2025	0.508
	7	2025	0.474
	10	2025	0.41
	11	2029	0.154
	12	2025	0.26
	13	2025	0.222
	14	2027	0.179
Residential heating	1	2025	0.4
	4	2025	0.39
	5	2030	0.161
	11	2025	0.423
	12	2025	0.322
	14	2028	0.166
Residential refrigeration	1	2025	1.073
	3	2025	0.209
	4	2025	0.755
	5	2025	0.558
	6	2025	0.366
	7	2025	0.86
	10	2025	0.429
	11	2025	0.651
	12	2025	0.801
	13	2025	0.392
	14	2025	0.324

**Table 3:** Load configuration results from 2021 to 2030.

Resource Type	Node	Time/(Year)	Capacity/(MW)
Electric vehicle	1	2021	2.129
	2	2026	1.288
	3	2022	0.536
	4	2021	1.856
	5	2021	0.843
	6	2021	0.876
	7	2021	2.571
	8	2029	0.206
	9	2028	0.211
	10	2021	1.23
	11	2021	0.756
	12	2021	0.986
	13	2021	0.682
	14	2021	0.565
Industrial heating	7	2023	0.22
Industrial refrigeration	1	2021	0.866
	7	2023	0.209
Commercial heating	1	2021	0.279
	4	2021	0.306
	5	2023	0.206
	7	2027	0.179
	10	2021	0.211
	12	2021	0.22
Commercial refrigeration	1	2021	0.426
	4	2021	0.591
	5	2021	0.508
	7	2021	0.474
	10	2021	0.41
	11	2029	0.154
	12	2021	0.26
	13	2023	0.222
	14	2027	0.179
Residential heating	1	2021	0.4
	4	2021	0.39
	5	2030	0.161
	11	2021	0.423
	12	2021	0.322
	14	2028	0.166
Residential refrigeration	1	2021	1.073
	3	2021	0.209
	4	2021	0.755
	5	2021	0.558
	6	2021	0.366
	7	2021	0.86
	10	2021	0.429
	11	2021	0.651
	12	2021	0.801
	13	2021	0.392
	14	2021	0.324

## Data Availability

Not applicable.

## Abbreviations

**Abbreviated Acronyms**	**Noun Full Name**
ARIMA	Autoregressive Integrated Moving Average model
*ACF*	Autocorrelation function
*PACF*	Partial autocorrelation function
*AIC*	Akaike information criterion
*BIC*	Bayesian information criterion
*ADF*	Automatic direction finder
